# First-Principles and Empirical Approaches to Predicting *In Vitro* Dissolution for Pharmaceutical Formulation and Process Development and for Product Release Testing

**DOI:** 10.1208/s12248-019-0297-y

**Published:** 2019-02-21

**Authors:** Nikolay Zaborenko, Zhenqi Shi, Claudia C. Corredor, Brandye M. Smith-Goettler, Limin Zhang, Andre Hermans, Colleen M. Neu, Md Anik Alam, Michael J. Cohen, Xujin Lu, Leah Xiong, Brian M. Zacour

**Affiliations:** 1 Small Molecule Design and Development, Eli Lilly and Company, Lilly Technology Center North, B302, Drop 3210, Indianapolis, Indiana 46285 USA; 2 Drug Product Science and Technology, Bristol-Myers Squibb, New Brunswick, New Jersey 08903 USA; 3 Merck & Co., Inc., Kenilworth, New Jersey 07033 USA; 4 Analytical Research and Development, Pfizer Inc., Groton, Connecticut 06340 USA; 5 Global Chemistry and Manufacturing Controls, Pfizer Inc., Groton, Connecticut 06340 USA

**Keywords:** drug dissolution, empirical modeling, first principles, *in vitro* dissolution, modeling and simulation (M&S)

## Abstract

This manuscript represents the perspective of the Dissolution Working Group of the International Consortium for Innovation and Quality in Pharmaceutical Development (IQ) and of two focus groups of the American Association of Pharmaceutical Scientists (AAPS): Process Analytical Technology (PAT) and *In Vitro* Release and Dissolution Testing (IVRDT). The intent of this manuscript is to show recent progress in the field of *in vitro* predictive dissolution modeling and to provide recommended general approaches to developing *in vitro* predictive dissolution models for both early- and late-stage formulation/process development and batch release. Different modeling approaches should be used at different stages of drug development based on product and process understanding available at those stages. Two industry case studies of current approaches used for modeling tablet dissolution are presented. These include examples of predictive model use for product development within the space explored during formulation and process optimization, as well as of dissolution models as surrogate tests in a regulatory filing. A review of an industry example of developing a dissolution model for real-time release testing (RTRt) and of academic case studies of enabling dissolution RTRt by near-infrared spectroscopy (NIRS) is also provided. These demonstrate multiple approaches for developing data-rich empirical models in the context of science- and risk-based process development to predict *in vitro* dissolution. Recommendations of modeling best practices are made, focused primarily on immediate-release (IR) oral delivery products for new drug applications. A general roadmap is presented for implementation of dissolution modeling for enhanced product understanding, robust control strategy, batch release testing, and flexibility toward post-approval changes.

## BACKGROUND

Orally administered solid dosage forms (tablets and capsules) constitute a large fraction of pharmaceutical products. These formulations are designed to release the active pharmaceutical ingredient (API) through the patient’s gastrointestinal (GI) tract in a prescribed manner. Understanding the *in vivo* mechanism of API release and absorption is a key objective to streamline and optimize the development of orally administered drug products. Dissolution testing is an *in vitro* laboratory performance test that assesses how efficiently a drug is released from its dosage form. During drug development, dissolution profiles have been used to understand the impact of formulation composition and process parameters on the *in vitro* release of API. Dissolution testing also plays an important role in the context of science- and risk-based process development, validation, evaluation of post-approval formulation changes to drug product quality, assessment of bioequivalence, and as a surrogate for *in vivo* drug release. In manufacturing, *in vitro* dissolution has been used routinely as a quality control (QC) release test to ensure batch-to-batch manufacturing consistency or quality. It has become an integral part of regulatory filings worldwide, with the expectation to serve as a QC tool to detect critical quality attribute (CQA) changes that affect *in vivo* release leading to exposure (i.e., bioperformance). It is also part of regulatory requirements to apply for a waiver of *in vivo* bioequivalence studies based on predictive *in vitro/in silico* methods (post-approval changes, new strengths, formulation modifications) (1–5).

Predictive dissolution modeling is an emerging methodology defined as the ability to mathematically generate a time profile of the dissolved amount of an API based on information regarding material properties, dissolution method conditions, formulation composition, and process parameters. While both *in vivo* and *in vitro* dissolution can be simulated and predicted, the focus of this paper is to document best practices for *in vitro* dissolution modeling. To the authors’ best knowledge, *in vitro* dissolution modeling in pharmaceutics has mainly been used for (1) early-stage formulation development or (2) real-time release testing (RTRt) in manufacturing.

The first usage category, dissolution modeling of *in vitro* behavior during early formulation and process development, leverages modeling exercises and targeted experimentation to identify critical material attributes (CMAs) and critical process parameters (CPPs). It is then used to screen the best plausible formulation for a robust operational design space and patient performance. Performing a first-principles-based comparison of dissolution performances can speed target formulation development or explain differences in *in vivo* behavior (6). It can facilitate formulation and process development, improve product quality, and reduce laboratory dissolution testing through enhanced product and process understanding.

In the second category, predictive *in vitro* dissolution modeling uses process understanding and input of CPPs and CMAs and/or real-time data to support RTRt in continuous or traditional batch process manufacturing (7–10). It enables commercial release via leveraging data collected both off-line and real-time (i.e., at-line and in-line) throughout a manufacturing process to predict a dissolution profile, rather than basing the release decision upon product testing conducted after completion of the manufacturing process. Thus, it can minimize or eliminate destructive testing of product tablets and speed material release.

Although the importance of *in vitro* dissolution modeling to traditional dissolution method development, RTRt, and product release is understood, literature review showed very few examples of predictive dissolution models being used in a QC environment and even fewer in real-time release situations. More examples are found in the formulation and process development space, but even in these cases there is no standard approach that has been adopted across the industry. With the motivation of filling this gap, members of the Process Analytical Technology (PAT) and the *In vitro* Release and Dissolution Testing (IVRDT) focus groups (FG) within the American Association of Pharmaceutical Scientists (AAPS) started a joint effort to present the experiences, practices, and thoughts on the topic. These FGs organized a series of conference symposia, including a structured seminar (with presentations from Bristol-Myers Squibb, Eli Lilly, Merck & Co., Inc., and Vertex) to share current applications of predictive *in vitro* dissolution throughout the product development cycle and to show examples of approaches used to model and predict *in vitro* dissolution. A debate session on the topic was also hosted at the 2016 AAPS Annual Meeting (11), and a short course was organized at the 2017 AAPS Annual Meeting (12) to share and promote the learnings. Discussions with the International Consortium for Innovation and Quality in Pharmaceutical Development (IQ) dissolution working group led to the authoring of this paper to share knowledge and experiences in developing dissolution models.

The purpose of this paper is to show recent progress in the field of *in vitro* predictive dissolution modeling, as well as to provide recommended general approaches to developing *in vitro* predictive dissolution models for both early- and late-stage formulation/process development and batch release. Two industry case studies of current approaches used for modeling tablet dissolution are presented. These include examples of predictive model use for product development within the space explored during formulation and process optimization, as well as of dissolution models as surrogate tests in a regulatory filing. A review of an industry example of developing dissolution model for RTRt and of academic case studies of enabling dissolution RTRt by near-infrared spectroscopy (NIRS) is also provided. These demonstrate multiple approaches to developing data-rich empirical models in the context of science- and risk-based process development to predict *in vitro* dissolution. Recommendations of modeling best practices are also provided, focused primarily on immediate-release (IR) oral delivery products for new drug applications. The intent of this paper is not to proscribe specific dissolution conditions (e.g., biorelevant media (13)) or target performance (e.g., clinically relevant specifications (14), which has been the focus of a recent white paper by the IQ Dissolution Analytical working group). Rather, the approaches outlined herein are designed to be applied independently of or in tandem with prior guides (13,14), dependent on the needs of a given project at its different stages of development.

The authors also hope to increase communication with regulatory authorities, working toward establishing an appropriate framework and acceptance criteria for the use of dissolution models in future regulatory submissions. *In vivo* predictive dissolution (an important part of predicting drug product *in vivo* performance) and the integration of dissolution profiles into physiology-based pharmacokinetic (PBPK) models are outside the scope of this paper.

## COMMON PRACTICES IN DEVELOPING *IN VITRO* PREDICTIVE DISSOLUTION MODELS

**Fig. 1:**
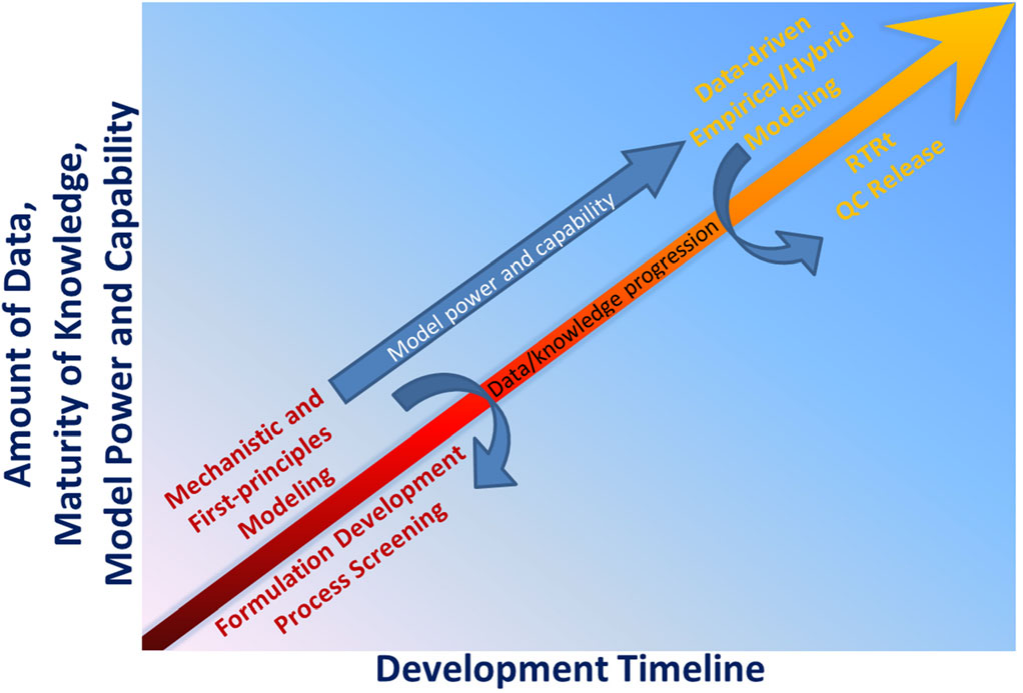
Description of the progression and use of dissolution modeling across a drug product development timeline

The development of predictive *in vitro* dissolution modeling applies the same principles as that of traditional experimental dissolution methods, with the same criteria for rejecting non-bioequivalent batches (i.e., dissolution behavior shown to correspond to an unacceptable deviation in expected bioperformance). In development of predictive dissolution models, empirical and first-principles-based approaches have been documented for a range of intended purposes. Combinations of the two, such as the use of first-principles-derived parameters as inputs for an empirical model and vice versa, are also common. Here, we categorize modeling approaches based on the type of model used for decision-making, regardless of the input source. For example, a hybrid model using first-principles-determined parameters as inputs for an empirical model for decision-making is considered as an empirical modeling approach, and vice versa. Figure 1 provides a cartoon description of the progression and use of dissolution modeling across a drug product development timeline. With project progression, as the amount of data and maturity of knowledge increase, so do the model capabilities and predictive power. Early in development, with a dearth of data, first-principles-based models are created to aid in formulation development and process screening. With increased data and knowledge, these models inform or mature into data-driven predictive models to enable RTRt and QC testing.

### Quality Target Product Profile: Linking Material Attributes and Process Parameters to *In Vitro* Dissolution

**Fig. 2:**
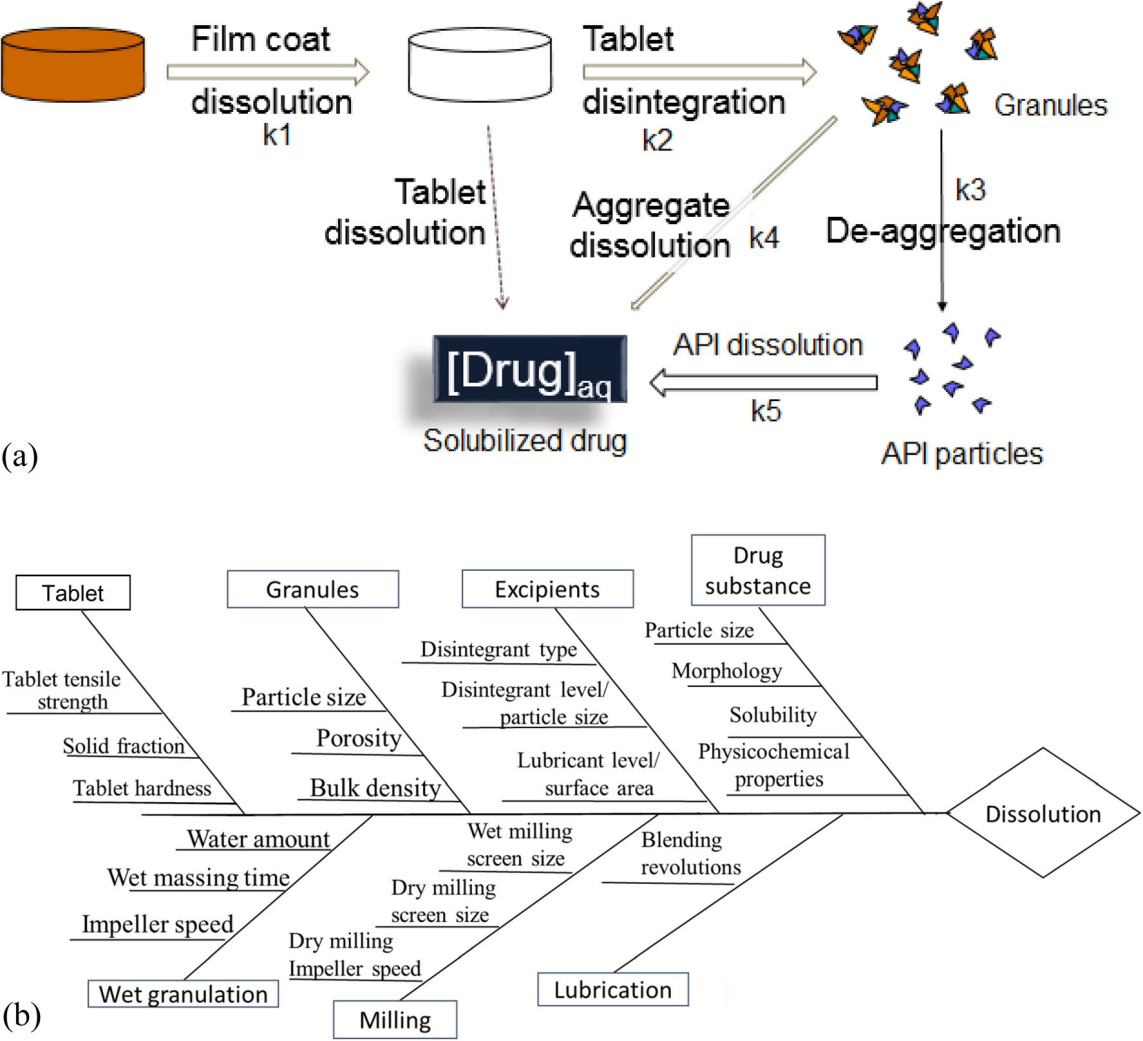
Rates and parameters affecting dissolution: **a** dissolution steps in an IR oral solid dosage unit and rate constant for each step (16); **b** example fishbone diagram of material attributes and process parameters affecting tablet dissolution in a wet granulation process

As a strategic drug product development process tool, a quality target product profile (QTPP) informs how the drug product is designed to best meet the needs of the patient. QTPP considers such factors as the dose(s), the dosing regimen, and other characteristics that might be important to the patient (e.g., unit size for pediatric or geriatric patients) (15). The QTPP, in conjunction with key pharmacokinetic characteristics of the API, helps define the particular formulation platform (e.g., an IR *vs*. modified release [MR] dosage form), as well as the choice of excipients in the formulation. A successful strategy for implementation of *in vitro* predictive dissolution starts with the QTPP in mind. The QTPP, which includes a target for how the drug is to be released *in vivo*, helps determine the CQAs that define the product quality. The QTPP would inform the formulation and process selection and, ultimately, the dissolution method development. The CQAs related to the product’s desired *in vitro* dissolution are of prime importance for consideration during dissolution modeling exercises. Before performing any modeling exercises, it is important to identify the CMAs and CPPs that are based upon physical and chemical properties of the API and of formulation and process selections, which, in turn, can be linked to the dissolution performance. For IR drug products, the Biopharmaceutics Classification System (BCS) provides a high-level guidance for CMAs based on said API properties (5), with Fig. 2a illustrating pertinent rates in a tablet dissolution profile (16). For an IR tablet, the typical rate-limiting steps are deaggregation into primary particles and dissolution of those particles (k2-k3 and k5 in the diagram), with BCS guidance around acceptable values of those overall rates relative to dosage unit strength.

Drug products that contain a highly soluble drug substance within the physiological pH range and are rapidly or very rapidly dissolving may be classified as BCS class I or III based on their absorption properties. A drug substance is considered highly soluble when the highest dose strength (defined as either the highest marketed dosage strength of an oral IR dosage form (5) or the highest single oral IR dose recommended for administration (17)) is soluble in 250 mL or less of aqueous media within the pH range of 1.2–6.8 at 37 ± 1°C. The distinction between the two definitions of dose strength is important and must be considered in dose selection when designing dissolution experiments or simulations to demonstrate BCS relevant dissolution characteristics or bioequivalence.

For drug products of BCS class I and III, the rate and extent of drug absorption is unlikely to be dependent on drug dissolution and/or GI transit time. Consequently, surrogate methods such as disintegration can replace dissolution to guide formulation development, following the International Conference on Harmonisation (ICH) procedure Q6a (18). United States Pharmacopoeia (USP) disintegration testing can be used in lieu of the dissolution test if the product is shown to meet a dissolution specification of ≥ 85% dissolved in 15 min (for BCS class III) or in 30 min (for BCS class I) across the physiological pH range. As an aside: on rare occasions, some excipients or drug-drug interactions alter GI motility, and dissolution rate of these components can affect bioperformance even for BCS class III or, in extreme cases, class I drug products. In such cases, the dissolution rates of the non-primary-API components may be more important than that of the API because of their direct or indirect physiological effects.

For those drug products of BCS class I and III that do not meet the above criteria, or for drug products of BCS class II and IV drug substances, i.e., characterized as poorly soluble, the dissolution profile must be carefully determined, as it could be the rate-limiting step for absorption (5). Thus, for these compounds, it is essential to thoroughly understand the factors that affect *in vitro* release. The factors chosen for the predictive modeling exercise can originate from either a risk-based approach or experimental data illustrating the impact of CMAs and CPPs on dissolution profiles. Experimental approaches often use scientifically rigorous design of experiment (DoE) and statistical analysis tools to model and understand dissolution profiles. This is typically done using, e.g., single-parameter fit followed by principal component analysis (PCA) or partial least squares (PLS) analysis, or a nonlinear model with an empirical fit. The intended purpose of these empirical approaches is to screen for the CMAs and CPPs for a follow-up DoE. For the risk-based approach, a fishbone diagram is often used as the first step to link CMAs and CPPs to the rate-limiting step(s) for dissolution. Figure 2b presents an example of such a fishbone diagram for a hypothetical process, showing some potential CMAs (e.g., material properties of API, excipients, granules, or tablet) and possible CPPs (e.g., settings in an established process for granulation, milling, or lubrication blending). If a risk-based approach is chosen, it is recommended to demonstrate enough dissolution variability from historical data to allow subsequent modeling algorithms to provide sufficient discriminating power.

### Dissolution Method Development

Development of a predictive dissolution model requires the availability of a dissolution method against which the model can be verified. An early-stage dissolution method can be developed based on API physicochemical properties and dissolution parameters selected to discriminate for anticipated critical attributes. At this stage, as the formulation and process are still evolving, surrogate methods such as disintegration may be adequate until it is understood which material attributes and process parameters are critical to dissolution. During late-stage product development, a QC method with clinical relevance is desirable if dissolution is shown to be rate-limiting for pharmacokinetic (PK) performance. Clinically relevant dissolution (14) may be achieved by evaluating CMAs, CPPs, and formulation variants in clinical PK studies, thus linking *in vitro* dissolution results with drug product *in vivo* performance. However, only a small number of clinical trials is typically conducted during the lifecycle of the product development. Thus, it is more common at this stage to develop a dissolution method that can discriminate for changes in CMA and CPP values and by setting a proper dissolution specification so that the *in vitro* dissolution performance of a commercial formulation does not deviate significantly from the pivotal clinical batches. The use of PBPK modeling is also encouraged to evaluate the clinical relevance of the dissolution method and to establish a dissolution safe space, as expected by health authorities.

When developing a dissolution method for model verification, it is helpful to first thoroughly understand the dissolution mechanism (as illustrated in Fig. 2a), particularly to determine which individual rate processes (e.g., surface dissolution, diffusive mass transfer, tablet disintegration) are rate-limiting for overall dissolution. A dissolution method should be designed to discriminate for those factors that impact the rate-limiting processes contributing to dissolution. Discriminating capability of the dissolution method is a key element that health authorities seek during review of the chemistry, manufacturing, and controls (CMC) section of new drug applications (NDA). Generally, the selected dissolution method and/or model should demonstrate discriminating ability for drug product manufactured under target conditions *vs*. the drug products that are intentionally manufactured with meaningful variations (e.g., ± 10–20% change to the specification limit/target value) for the most relevant manufacturing variables and material attributes (e.g., drug substance particle size, compression force, tablet hardness). While a link between dissolution and clinical performance would ideally be established, there is currently no standard approach or regulatory framework for the industry on how to develop and implement clinically relevant dissolution modeling (19). If discriminating capability of the method is demonstrated on the most relevant material attributes and manufacturing variables, the method can be considered as a QC tool to be used in commercial and stability testing to ensure drug product quality. The predictive model should be calibrated against such a dissolution method; thus, the discriminating ability of the predictive model hinges upon that of the dissolution method. A less discriminating dissolution method is expected to result in a less capable predictive dissolution model.

### First-Principles Approaches

**Fig. 3:**
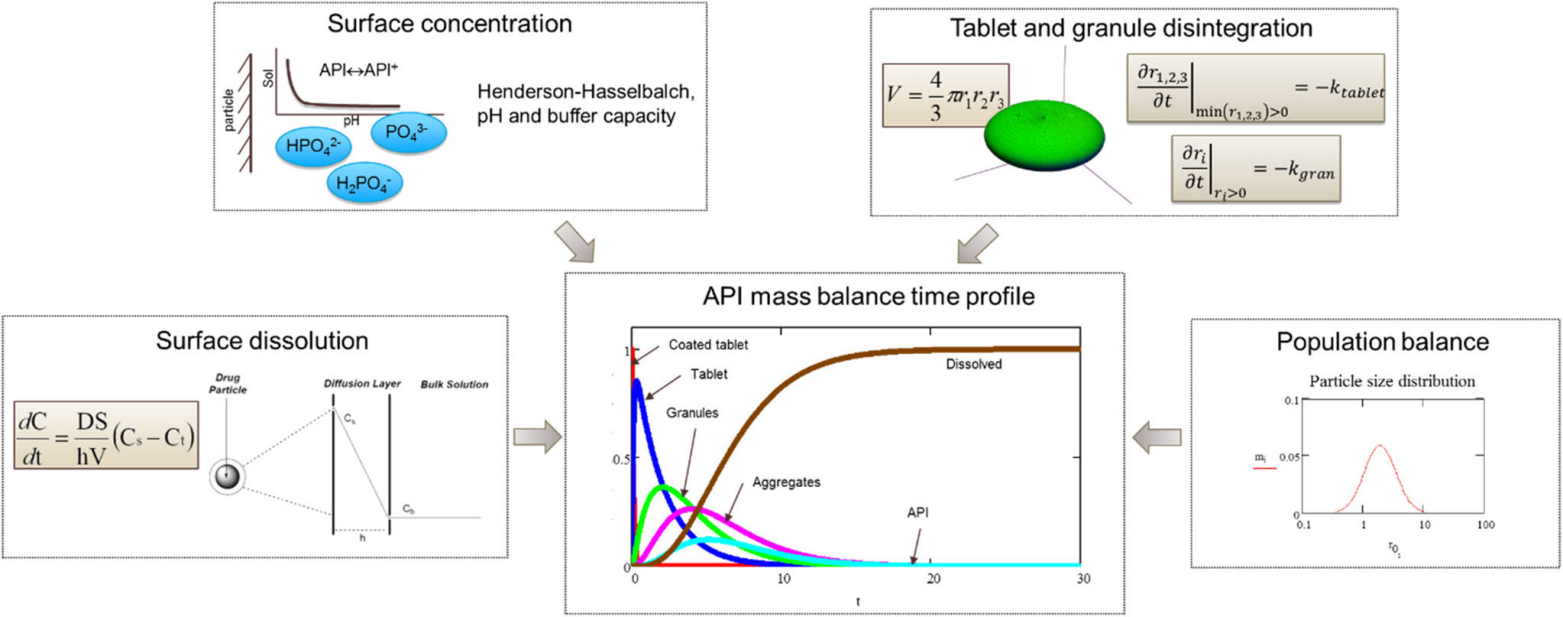
Example of first-principles components informing a dissolution model and predicting a dissolution time profile

First-principles approaches to predictive dissolution modeling can be useful very early in drug product development, even before a single dissolution experiment is performed. Physical models of dissolution-contributing phenomena (e.g., hydrodynamics, solubility *vs*. pH), coupled with readily measured physical characteristics of a drug substance (e.g., intrinsic solubility, pK_a_, average particle size), provide the ability to rapidly establish a dissolution method for product development (e.g., Fig. 3 (20)). Additionally, bottom-up dissolution prediction can provide early guidance on dosage and on particle size without necessitating dissolution testing of drug product. A subsequent section of this paper (case study 1) details a case study demonstrating these approaches, following the guidelines for implementation given at the end of this section.

The first-principles study of dissolution profiles of chemicals has begun 120 years ago, tracing its roots to Arthur Noyes and Willis Whitney’s seminal publication in 1897 (21). This work characterized dissolution as a 1st-order rate process, dependent only on the material solubility and a rate parameter *k*. At sink conditions, this model further reduces to a 0-order rate. These 0th- and 1st-order kinetics are the most basic mechanistic descriptions of dissolution, but they are at the heart of all first-principles dissolution models.

Over a century of research has gone into expanding and augmenting the Noyes-Whitney equation, very aptly reviewed by Dokoumetzidis and Macheras (22). Most of the work has been done on deconvolution of the 1st-order dissolution rate constant *k* into molecule- and media-dependent parameters, adding further dependencies and interdependencies, introducing particle population models and geometry characterizations, and enabling greater process understanding. As our ability to measure and characterize material properties grew, so has our understanding of particle dissolution. Other mathematical relationships have also been explored (23), including empirical equations (e.g., Weibull) and hybrid models that use understanding of the dissolution mechanism to parametrize empirical equations.

One of the major advances in first-principles dissolution theory, by Walther Nernst (24) and Erich Brunner (25), tied dissolution to Fick’s second law of diffusion, positing the following model, now known as the Nernst-Brunner equation (Eq. 1):

1 $$ \frac{dc}{dt}=\frac{DS}{Vh}\left({c}_s-c\right) $$

where *c* is the concentration of the species of interest, *D* is its diffusion coefficient across the diffusion layer, *S* the surface area available to dissolution, *V* the volume of dissolving media, *h* the thickness of the diffusion layer, and *c*_*s*_ the surface concentration of the dissolving material (often assumed to be equivalent to solubility at local surface pH unless surface kinetics are additionally considered, as discussed below).

The parameter of the available dissolution surface area *S* depends on the particle (or granule) size distribution (26) and shape factor (27). It is also a function of time based on particle population balance (28) and on drug product disintegration, through which the primary particles are made available to be dissolved by the medium. An excellent review of drug tablet disintegration theory and modeling has been performed by Markl and Zeitler (29). Because surface area is a function of both disintegration kinetics and of the level of dissolution, it leads to the dissolution rate no longer being 1st-order (or 0th-order at sink conditions), adding the mathematical complexity of reactions-in-series.

Each of the above parameters has also had decades of research dedicated to its refinement. For example, models have been developed to estimate the diffusion coefficient from solute and/or dissolution medium properties (30–33). Similarly, solubility has been modeled via the Hendersen-Hasselbalch equation for ionic dissociating species (34,35), modified for medium ionic strength (36–38), and using quantum chemical calculations to predict intrinsic solubility (39). The diffusion boundary layer has been modeled as a function of dissolving particle size (40), particle shape (27), medium/setup hydrodynamics (41) and volume of confinement (42), and polydisperse, polymorph drug particles (43), which also makes it a time-variable coefficient and not a constant. The overall intrinsic dissolution rate has also been calculated from first-principles analysis of molecular structure and crystal predictions (44–47), as well as from analysis of the interaction of solubility, surface kinetics, and diffusive mass transfer (48). Work has been done to derive from first principles and to parametrize the disintegration rate of an IR tablet and the concomitant release of API particles for dissolution (49–51) and to develop formulation-predictive dissolution (52).

All of these variables are important to consider in varying degrees and at different stages during drug product development: in selecting dissolution methods for prototype formulation, process screening, and QC release test, in understanding drug product *in vivo* performance, and in establishing *in vivo*/*in vitro* correlation (IVIVC). Each variable’s importance may be limited by the selection of materials and/or environment. For example, a given solute and dissolution method fixes the dissolution volume *V*. In that volume of a specific medium at a given agitation rate, the values of the aforementioned variables are almost entirely dependent on the concentration of the solute (API) and its ratio to some of the excipients. Specifically, surface concentration (*c*_*s*_) is affected by pH modifiers and surfactants. Boundary layer thickness of a given particle size (*h*(*t*), since particle size is a function of time) can be affected by viscosity-modifying surfactants/wetting agents. Diffusion coefficient across the boundary layer (*D*) may be changed by viscosity modifiers and micelle-formers. Thus, these parameters are functions only of drug product composition (and its deviation from target). Adherence to target composition can be ensured through precise control of process parameters such as the amount of raw material dispensed and mixing time and verified real-time by spectroscopic methods such as tablet NIRS, as demonstrated in literature (10,53–68).

Many academic researchers have discussed and developed methodology for the use of first-principles predictive dissolution modeling in the pharmaceutical industry (23,69). However, there are few documented cases in literature of industrial applications of such methodology, with the exception of a few notable examples (6,27,70,71). A novel example of first-principles dissolution modeling for pharmaceutical method development is discussed in more detail in the first case study below. The following general systematic approach is suggested for first-principles dissolution model development.

1. Establish molecule-specific properties contributing to a dissolution rate: primary particle density, solubility across the physiological pH range, diffusion coefficient (ideally, measured directly or through intrinsic dissolution rate measurement; alternatively, fitted to a dissolution profile of well-characterized powder free of formulation effects), boundary layer thickness as a function of particle geometry and density (if not using the Hintz-Johnson model (40)).
2. Select a test setup (e.g., USP apparatus II with 900 mL of media) and dissolution medium that would provide a dose number (dose mass divided by test volume divided by solubility) of, ideally, 0.35–0.9. This is important because it allows the dissolution profile to display contributions by both the small particles/high specific surface area material (in the initial slope) and the opposite end of the distribution (the final part of the profile). Too low of a dose number can mask the contribution of fines, and a dose number above 1 would lead to larger particles (or mass-transfer-limited material) to remain undissolved, masking their contributions to the profile and their effects on formulation changes. If simple buffers or simulated gastric/intestinal fluids do not provide sufficient solubility, consider fractional doses (e.g., scaled down tablets, tablet fractions) or media with solubility enhancers (e.g., surfactants).
3. Determine or adjust the values of parameters affected by media composition (i.e., diffusion coefficient, solubility) and apparatus configuration (i.e., boundary layer thickness).
4. Characterize the API surface area distribution (for pure API, by measuring particle size distribution [PSD] and morphology; for alternatives such as spray-dried dispersion or deposition on solid support, by measuring or estimating thickness of API-containing material layers and their distribution). Model the rate of change of surface area (and its distribution) with dissolution progression, as pertinent to the specific geometry of the API-containing solids.
5. Model the dissolution of unformulated API in the selected medium by combining the measurements of the driving-force-related properties with the characterization and model of available surface area *vs*. time. Establish the “pure” (unformulated API) dissolution profile as the baseline.
6. Validate this model by performing the unformulated API dissolution experiment, being careful to avoid phenomena such as barriers to wetting or particle sedimentation. Adjust any model assumptions that may have led to deviation of the model from experimental observation. Alternatively, use this dissolution test to establish a scaling factor for the driving force to account for difficult-to-model geometries (e.g., wide aspect ratio distribution of crystals, diffusion of API out of porous support).
7. Use the model to demonstrate effects of process, API form, and formulation changes, such as acceptable particle size range, solubility change due to change in polymorphic form or salt, risks of poor disintegration or over-granulation (e.g., via apparent surface area reduction or retardant effects), effect of surfactants in formulation on dissolution, opportunities for mitigation of non-ideal gastric pH through formulation additives, etc.
8. Demonstrate experimentally the behavior of formulated *vs*. ideal drug, and use the model to guide drug formulation prototyping to achieve target dissolution behavior.
9. If multiple prototype formulations with different dissolution behaviors are subsequently evaluated *in vivo*, use the *in vivo* results in conjunction with observed *in vitro* behavior to verify and correct the developed dissolution model and to establish clinically relevant dissolution specifications (14).

### Empirical Approaches

Empirical approaches documented to date are typically data-driven methods leveraging statistical and/or chemometric regression algorithms, such as generalized linear models and PLS. Due to the nature of the empirical approach, an empirically predictive *in vitro* dissolution model is dependent upon a traditional dissolution method. These predictive models are used to forecast the dissolution profiles either directly (i.e., release level at specific time points or time to reach a specified release) or through predicting values of coefficients for fits to functional forms of dissolution profiles. In contrast to the first-principles approaches, which are frequently used to gain product and process understanding, empirical methods are generally intended more for release testing. This is likely due to the increasing use of automated data historian and PAT data warehousing packages in the pharmaceutical industry. Because this approach is data-driven, the development of an empirical predictive dissolution model typically occurs during late-phase development program and follows the “lifecycle approach,” where the development of a dissolution model is concurrent with formulation development and process optimization. The details of applying a lifecycle approach to develop a sensitive and robust chemometric model for dissolution shall be consistent with the general guidelines for developing a chemometric model, which can be found elsewhere (72,73). Exploring the formulation and process knowledge space provides an opportunity to understand the impact of various formulation and process variables on dissolution, which also establishes a foundation for the subsequent model-building exercise. The predictive model is then expected to serve as a surrogate for product dissolution testing within the space explored during formulation/process optimization. However, the use of the predictive model as a test surrogate in a regulatory filing is not always the end goal of such a modeling exercise. Modeling efforts could also be leveraged to improve process understanding and to support product development (see case study 2).

Two types of data sources are often used in the empirical approach for dissolution modeling. The first type is off-line set-points and characterization data, such as individual or combinational variables of formulation (e.g., composition, component particle sizes, or molecular weights) and process conditions (e.g., compression force, blending time, granulation endpoint moisture content). The use of these parameters in building quantitative models of dissolution profiles/parameters has been a common practice during formulation and process optimization for a desired release profile for both IR and MR products (1–4,74–78). Multiple linear regression and response surface methodology are often used for that purpose. The second type is real-time data, often via the use of non-invasive analytical tools, collected at-line, on-line, and in-line during the manufacturing process. Examples include tablet weight and thickness, blend or tablet NIRS data, or final product visual imaging. Since the issuance of the PAT guideline by the United States Food and Drug Administration (FDA) in 2004 (7), the use of these tools (especially spectroscopy) has gained popularity. The most commonly used spectroscopic data is NIRS, given its sensitivity to both chemical and physical properties and its versatility to analyze samples of powder, ribbon, tablets, etc. (10,53–68). Although the majority of the referenced applications use only one of these data source types, uses of combinations of the two have gained popularity, at least in part due to enabling drug product release by leveraging real-time data collected throughout a manufacturing process. A literature review related to the topic of RTRt is provided in the later section of the paper.

Given the empirical nature of these dissolution models intended for release testing, the clinical relevance of these QC release method needs to be evaluated on a case-by-case basis based on the physical, chemical, and physiological properties of the drug product. The key here is to understand the relationship between dissolution and clinical relevance, which is often carried out via dedicated PBPK modeling and/or IVIVC and PK studies. In cases when a dissolution method is found to be clinically relevant, such a QC release method is expected to reject non-bioequivalent batches. In such cases, dissolution is considered the critical *in vivo* surrogate sensor as it is the only *in vitro* test that probes the extent and rate of *in vivo* release. For cases when no clinical relevance is observed for dissolution (e.g., dissolution is not the rate-limiting step of drug absorption), it is possible to establish a Normal Operating Range of the formulation and process conditions well within the design space explored during the formulation and process development. In those cases, a dissolution model (if established) can predict with confidence that every batch of product manufactured within that space would pass dissolution specification. The use of continuous manufacturing provides additional assurance compared to traditional batch process that such a Normal Operating Range is achieved within state-of-control in a consistent manner. In that scenario, it may no longer matter whether the predictive dissolution model leverages any real-time data, since at that point real-time collected data would only verify the entire manufacturing process as it affects expected dissolution and would no longer be a decision-making point. In such a case, the dissolution test shall be considered of low/medium risk. Thus, instead of developing a predictive dissolution model, mapping out a dissolution safe space is expected to provide regulatory flexibility and can potentially justify setting a wider specification given the safe space identified through formulation/process development and optimization and supported by justifiable PBPK evidence.

### The Dependent Variables

The dissolution models are typically used to forecast either (1) dissolution percentage at a specific time point (or the converse: time required to reach a specific percentage of release), or (2) a mathematical description of dissolution profiles with calculated or fitted coefficients (kinetics-driven exponential decay, Weibull profile, etc.). Less common model outputs have been used, such as spline fits for missing time points (although this can lead to non-physical profile values) or multiple-API interdependent release profile prediction. Discussion of these target outputs is out of scope of this paper; however, the methodologies and guidance herein can be readily extended to such advanced model uses.

Each approach has its own pros and cons and is best applied to different phases of product development. For a target release level at a fixed time point, both measured and predicted values have been reported. This is the most commonly used release specification and the easiest to evaluate and apply in a QC environment and under a manufacturing setting. However, it potentially ignores valuable information found by evaluating the entire profile. By contrast, the fitted or predicted full-profile target demonstrates systematic understanding of the formulation and process design space, since these profile-defining parameters are metrics containing aggregate information on the entire dissolution time profile. With a proper DoE and application of split plot designs to account for multiple sources of error, full dissolution profile prediction can be used to discriminate for design variables across multiple unit operations. Although a release specification on a fitted or calculated coefficient is not a typical regulatory-approved approach, it can provide guidance and flexibility in a post-approval change setting to justify which time point is to be used as the univariate release spec, even for interpolated time points. For instance, the highest discrimination power across a dissolution profile within a DoE may not be at a pre-defined time point, e.g., 43.7 min. The use of interpolated dissolution profiles based upon fitted/calculated parameters allows the release spec to be set at the time point with highest discriminating ability if that meets the intended purpose.

### Model Validation

A predictive *in vitro* dissolution model is dependent upon a traditional dissolution method. Thus, it is imperative that the reference method is suitably validated and is sufficiently discriminating to changes in dissolution-affecting CMAs and/or CPPs. Predictive *in vitro* dissolution method development is multifaceted given that calibration samples are subject to typical manufacturing variability. A robust model necessitates a calibration set with anticipated sources of variation that includes, but is not limited to, varying raw material attributes, different scales of production or material throughput rates, and instrument/equipment/environmental changes. It is of utmost importance that an independent validation set that spans the operational space defined by the calibration set be used for model validation. Achieving all these requirements in a single manufacturing campaign is improbable. In addition, model development and implementation should be viewed as a lifecycle approach where the rigor of validation is commensurate with the model’s impact on drug product quality (79). For example, the validation protocol of a model used for drug product release (i.e., a high-impact model) would require a more rigorous validation protocol in contrast to a model used during early R&D for formulation development (i.e., a low-impact model). Often, a low-impact model supporting development does not require additional validation.

Validation of a predictive dissolution model used as a surrogate for traditional dissolution testing (i.e., RTRt) is similar to the validation of the more well-established NIRS-based model for content uniformity release testing. For example, both are dependent upon the following: a reference method, data from validated sources, an understanding of variables and/or variable interactions affecting the response, a statistically sound and clearly defined sampling plan, an alternate method in the case of PAT equipment being unavailable, and a lifecycle management strategy. A potential difference is that a predictive dissolution model can include data from multiple in-process methods (e.g., granule PSD and moisture) that should themselves be validated. Validation elements specific to a predictive dissolution model include accuracy relative to the reference method and robustness. Linearity and accuracy can be demonstrated by the correlation coefficient R and the root mean square error, respectively, for an observed *versus* predicted fit of either (1) percentage dissolved at a specific time point or (2) dissolution profile nonlinear regression coefficients (59,64). To gauge model accuracy, a comparison of passing/failing acceptance criteria, the root mean square error of calibration and/or cross-validation, and the root mean error of prediction (using an independent validation set) can be reported. Furthermore, if the entire dissolution profile is predicted, an observed *versus* predicted similarity metric (e.g., f_1_, f_2_, or Mahalanobis distance) can be presented to indicate equivalency to the reference method (59,64,80). Demonstration of model robustness should leverage data collected throughout process and model development with designed sources of variability. Variations studied can be deemed insignificant to model performance (i.e., robust) using statistical probability testing. Material, process, and/or equipment variations that have a significant effect on the model should be rigorously managed via a defined model operating space and the development of model outlier diagnostics sensitive to such variations. Outlier detection methods are a common risk mitigation strategy in multivariate regression models. These are statistical tests (e.g., Hotelling’s T^2^ and residual variance) conducted to determine whether the analysis of a multivariate response using a calibration model represents a result outside the calibration space. Further details on validation can be found within the referenced FDA guidance for industry (79).

### Lifecycle Management

Model lifecycles must be appropriate to the phase of drug development in which they are applied. First-principles models established to support early formulation and process development are not necessarily expected to be used throughout the lifecycle of a drug product. Thus, lifecycle management mainly refers to models deployed to serve as surrogate tests for drug product release.

Two different deployment approaches are common in the field after a predictive dissolution model passes its validation for the purpose of RTRt. One approach is to use the validated model for batch release immediately from the first commercial campaign. The other is to perform, in parallel, both traditional dissolution testing and predictive dissolution modeling. After consistent agreement between the two methods, evaluated by appropriate statistical means (such as the sequential probability ratio test), the pharmaceutical manufacturer has the option to use the predictive dissolution model exclusively for the purpose of real-time batch release. Based upon prior successful experiences with predictive models for RTRt, it is highly recommended to gain pre-alignment with regulatory agencies regarding the choice of deployment approach.

If an empirical model predicts dissolution profiles of a pool of tablets rather than of individual tablets, regulatory agencies may request careful consideration of how and when to physically sample and test tablets. Physical testing in addition to predicted dissolution results may be needed to assure the uniformity of drug product performance within a manufacturing campaign. The prediction of a pool of tablets or of individual tablets is dependent on the nature of the inputs for such an empirical model. For instance, if an empirical model leverages d50 of API particle size and the compaction force as model inputs, it is likely that the predicted dissolution result is an averaged dissolution performance of the batch, not representative of individual tablets. For the predicted sample requirement, it has been proposed that the “sampling strategy” for product release follow the USP <711> Q + 5% rule for pooled samples (81). This rule predicates that a predictive model that uses averaged data will have 12 predictions per batch to meet the USP requirements. Thus, if the predicted average value fails the Q + 5% specification, additional physically sampled tablets from the batch are expected to be subject to traditional stage-3 release testing in addition to predicted results in order to meet the USP requirement. For a continuous manufacturing process, the principles of USP <711> stage 2 with stratified dissolution monitoring for RTRt is recommended (81). Dissolution should be predicted in several segments (e.g., 12) for each “batch” to confirm that the results comply with USP <711> dissolution stage-2 criteria.

A predictive dissolution model used for RTRt requires model maintenance and ongoing verification, regardless of whether it is deployed for batch or continuous process. Current good manufacturing practices (cGMP) quality procedure shall be in place to determine the triggers for model update and the frequency of ongoing verification. Controls must be in place to identify special causes of variation and common causes of variation with abnormal magnitudes or patterns. Continuous processing may offer earlier and more sensitive detection of such variations for model updates compared to batch processes. Periodic verification against a reference dissolution method is typically done to assure the validity of common causes of variation. Special-cause-triggered verification may take place on as-needed basis, such as a change in excipient vendor. The trigger for model update often relies on multivariate diagnostics values (such as Hotelling T^2^ and residual values) indicating that the process or material changes have a significant potential to impact drug product dissolution. Control limits shall be in place for these diagnostic values to keep the method in a validated state. If a model update is needed, a protocol-based revalidation shall be performed according to its level of impact in order to bring the predictive model back to its validated state.

Post-approval changes can be aided by dissolution modeling as well, especially if validated dissolution models were part of the original submission. The modeling approach and utility of dissolution modeling for post-approval changes is highly dependent on the nature and level of the change. Mechanistic understanding of the dissolution phenomena that is used in prototype development and formulation selection can also be used to predict the effects of post-approval changes with minimal, targeted experimentation. For manufacturing release and QC, validated dissolution models for batch release or RTRt and/or model-based *in vitro* dissolution methods can be used following post-approval changes if it can be shown that the model space encompasses the process post-change. If the change positions the process outside the limits of existing release models or methods, then the process understanding gained through establishing the models can also be used to guide experiments for reestablishing and revalidating the models/methods with minimal experimental burden. In these cases, dissolution models can be powerful tools to minimize *in vivo* and *in vitro* experimental burden to support post-approval changes.

## CASE STUDIES

### Example of First-Principles Approach in Early Development

Drug substance A (DS-A) is a weakly basic, highly permeable compound (effective permeability *P*_eff_ of 2.18 × 10^−4^ cm/s) with a p*K*_a_ value of 7.6 and an intrinsic solubility of 0.15 μg/mL. It thus exhibits a solubility difference of |3 orders of magnitude (from 0.01 to > 10 mg/mL) across the range of typical gastric pH values. This compound was prepared as a tosylate salt, with a salt solubility of 1.8 mg/mL, and a pH_max_ of 3.5. The precipitation kinetics of DS-A in aqueous media are exceedingly slow, maintaining supersaturation at ratios orders of magnitude above 1. Therefore, drug product design was intended to ensure consistent dissolution of the drug product across the range of gastric conditions and to rely on maintained supersaturation to drive permeation and absorption of the drug substance in the intestinal tract. To that end, first-principles modeling and simulation were used to design targeted experiments to develop a dissolution method that best differentiated among prototype formulations. This method was then used in conjunction with the first-principles-based models to develop a tablet formulation for optimal dissolution performance.

**Fig. 4:**
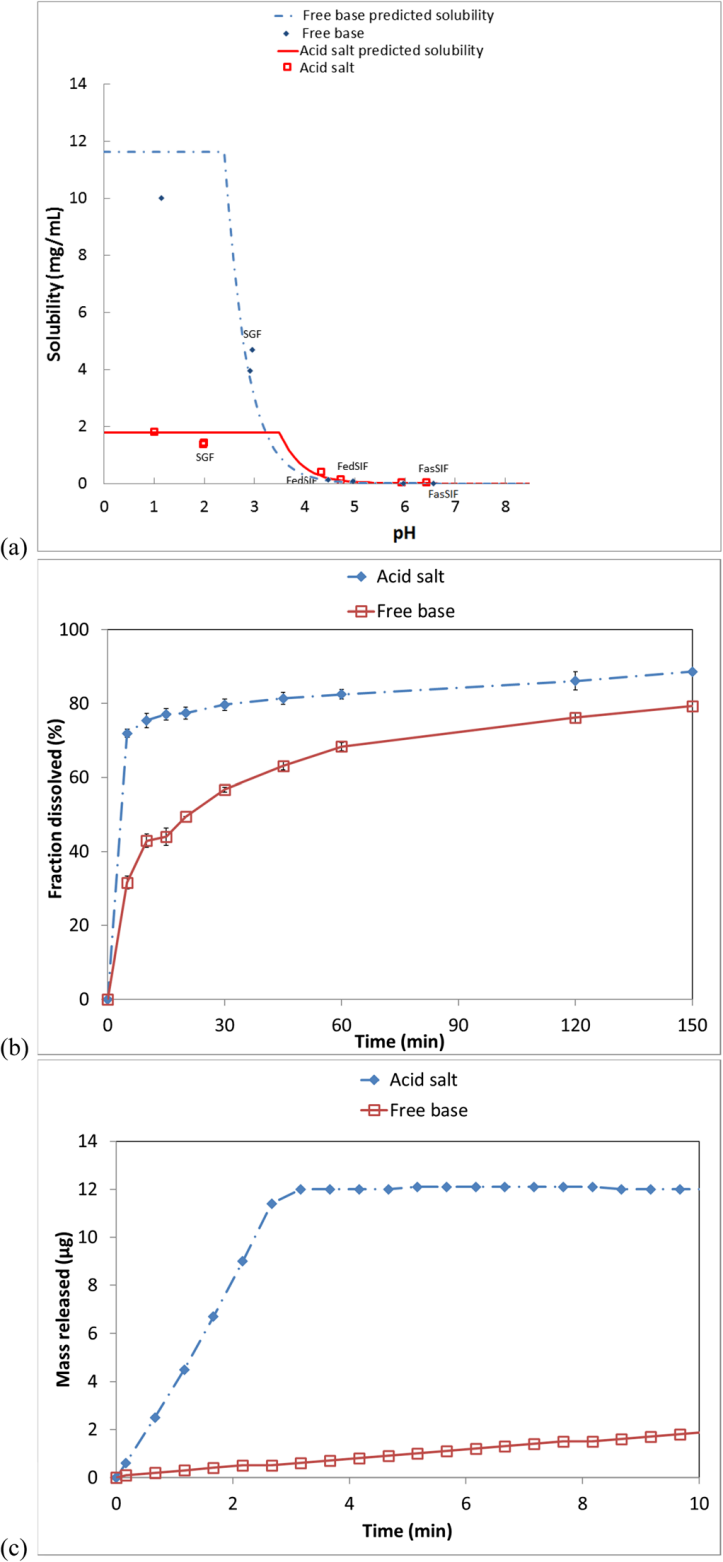
Comparison of free base and salt of DS-A: **a** solubility *vs*. pH; **b** dissolution profiles of 12 mg strength tablets in pH 4.5 50 mM acetate buffer, **c** intrinsic dissolution in pH 6.5 phosphate buffer

For an ionizable compound such as DS-A, the saturation concentration (*c*_*s*_ in Eq. 1) at the particle surface is often different from that in bulk liquid in many buffer media due to the contribution of the solute itself (the charged API species as well as the acid counterion) to the ion balance (82) (see Fig. 4a for solubility differences between salt and free base in the physiological pH range). Therefore, for a salt of a basic compound in a medium above pH_max_ in the bulk, the particle surface pH may be much lower and equilibrium solubility at particle surface may be much higher than those of the free base due to the contribution of the acid counterion to the local pH. This leads to a more rapid dissolution of the salt than of the free base at the same conditions. In fact, this was observed in tablets made with similar PSDs of free base and tosylate salt of DS-A using equivalent tablet formulations, as exemplified for 12-mg-strength (free base equivalents) tablet dissolution in pH 4.5 acetate buffer (50 mM), shown in Fig. 4b.

Figure 4b also makes it apparent that there is a sharp inflection in the salt dissolution profile. This inflection was confirmed when evaluating the intrinsic dissolution rates (IDR) of salt and free base in a medium with pH > pH_max_ (Fig. 4c). Spectroscopic measurements (NIRS, Raman, and X-ray diffractometry [XRD]) of the IDR compacts showed that the surface of the salt compact was composed of free base after exposure to the dissolution medium and that this only extended to a very small depth. It was hypothesized that the tosylic acid, due to its very high solubility in water, was able to dissolve from the crystal structure and to diffuse away more rapidly. The outer surface of the particle then reformed as API free base crystal. Subsequently, the surface of the particle then exhibited characteristics of the free base for the remainder of the particle’s existence in the dissolution medium. This inflection appeared to occur consistently after ~ 3 min following the powder’s exposure to the medium, regardless of the medium pH (above pH_max_).

To aid in drug product development of an oral IR tablet, a dissolution model predictive of in vitro-observed behavior was developed. Particles were assumed to be spherical, with a distribution as measured by laser light scattering. The dissolution of a single particle was modeled using the Hintz-Johnson approach (40) (the Nernst-Brunner equation, with *h* equal to the lesser of particle radius or 30 μm). The solubility of API at particle surface was calculated using the Dressman method (82) by assuming saturation in the specific buffered medium, limited by diffusion rates of individual solute species. For the salt, the assumption was made that at the surface (source), there is also one stoichiometric equivalent of the tosylic acid (total of dissociated and undissociated) relative to the API, thus reducing the pH at particle surface and enhancing solubility. Particles of the DS-A tosylate salt were modeled to behave based on the salt properties for 3 min following their being wetted by the medium. Subsequently, they were then modeled to behave as if they were free base particles. Thus, two separate populations of particles were established, the dissolution of both contributing to the bulk concentration of the medium. The two dissolving population balances were treated in the standard manner (83), with the added complication that the particles in the salt population also had an age distribution orthogonal to size distribution. Any particles that reached the age of 3 min were transferred from the salt population to the free base population.

Dissolution from a capsule was modeled by assuming that API particles remain dry (unexposed to the dissolution medium) prior to release and that the release of solids from the capsule shell follows a Weibull distribution. The values of the shape and scale of the distribution were fitted to experimental data, with a single set of values fitted across multiple dissolution media.

**Fig. 5:**
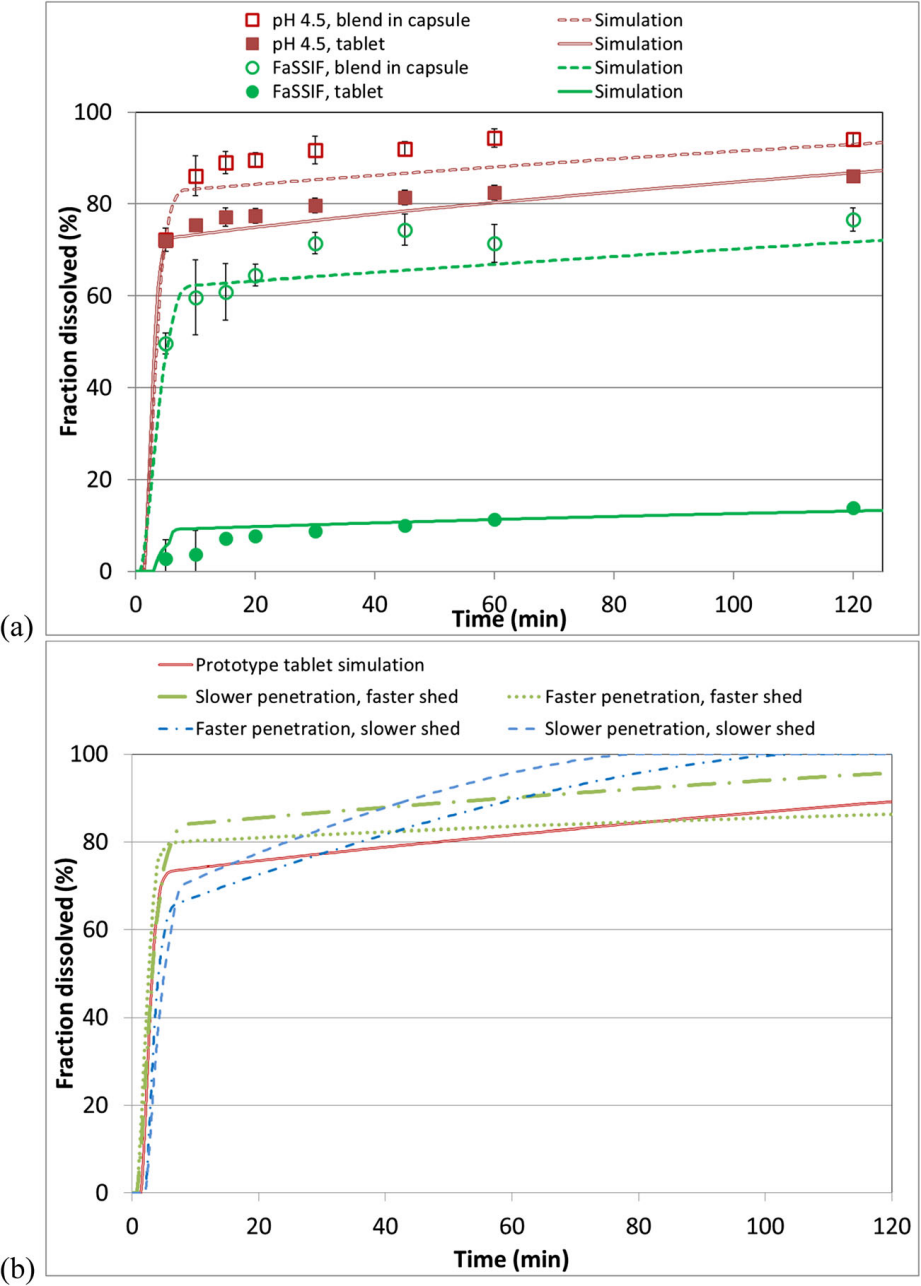
Experimental and simulated dissolution profiles of DS-A: **a** tablet *vs*. blend-in-capsule with identical compositions, in FaSSIF and pH 4.5 50 mM acetate buffer, and **b** tablets in pH 4.5 50 mM acetate buffer, with varying media penetration rate into the tablet and the time required for wetted material to be shed from the tablet

Dissolution from a tablet was modeled by fitting the real tablet volume to an ellipsoid model using the tablet’s measured solid fraction and average density. The API was assumed to be distributed evenly throughout the tablet. Dissolution media was then assumed to penetrate the tablet at a fixed velocity (tablet depth *vs*. time), with material leaving the tablet after a specified wetting time. The media in the interstices of the tablet were assumed to saturate with API. The drug particles were treated as being exposed to the media for the duration of the wetting time before being released to bulk media to continue their dissolution. The values of media penetration velocity and the wetting time before release were fitted to experimental data. The wetting time before release was assumed to be same across multiple dissolution media. However, two different media penetration velocity values were fitted; one for surfactant-containing media (fasted-state simulated intestinal fluid [FaSSIF]) and another for non-surfactant media. Figure 5a shows experimental data and simulated dissolution profiles in FaSSIF and in pH 4.5 50 mM acetate buffer for early prototype tablets and for tableting blend placed into capsule shells. At higher pH, a very high dependence of dissolution rate on dispersion ability is observed. However, at pH above 5.5, free base cannot be fully dissolved in 900 mL for tablet strengths under evaluation. The pH 4.5 acetate buffer was selected as a medium able to provide very strong differentiation for tablet behavior while still allowing both forms of API to fully dissolve at strengths of interest (albeit very slowly for the free base). While this method was not intended for use as a QC method because it was not yet clear at this stage whether it discriminates for any clinically meaningful differences, it was useful for prototyping and optimizing formulation development.

**Fig. 6:**
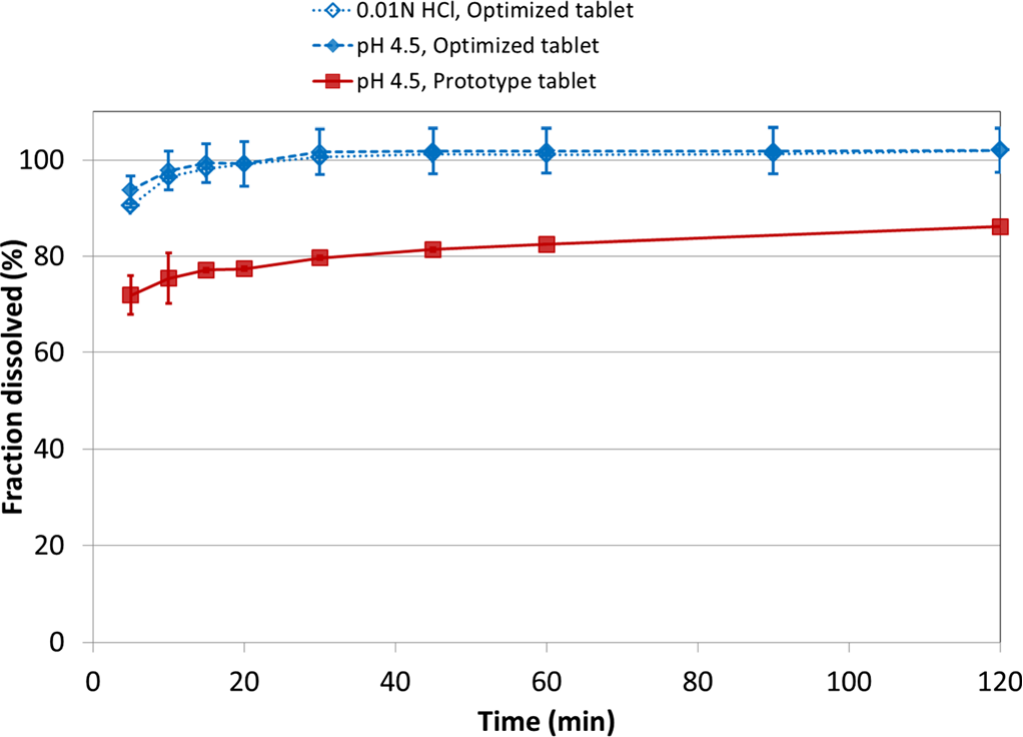
Dissolution profiles of DS-A prototype tablet in pH 4.5 50 mM acetate buffer and optimized tablet in 0.01 N HCl and pH 4.5 50 mM acetate buffer

By exploring time trajectories of several variables within the model (e.g., PSDs of the two populations, bulk and surface concentrations), it was possible to determine the first-principles causality between dissolution rates of the two populations and the properties of the drug product. Figure 5b demonstrates the variation in simulated dissolution profiles of the tablet formulation in a virtual DoE where the media penetration rate into the tablet was increased or decreased by a factor of 1.25 while the wetting time for material to shed from the tablet was raised or lowered by 50%. This allowed for an optimization routine that led to the redevelopment of the tablet. Reducing drug load by a factor of 3 (increasing tablet size), increasing solid fraction, and increasing the level of disintegrant led to a tablet with slower media penetration, faster shedding once wetted, and an API release profile that resulted in optimal dissolution behavior. Figure 6 shows the dissolution profiles in pH 4.5 acetate buffer of the initial prototype tablet (same as shown in Fig. 5a) and of the optimized tablet, as well as that of the optimized tablet in 0.01 N HCl. It is clear that the dissolution of the optimized tablet is far improved over that of the prototype, and the overlap of dissolution profiles in 0.01 N HCl (pH 2 medium) and pH 4.5 medium provides confidence of more uniform dissolution behavior across a much broader range of patient gastric conditions.

### Example of Empirical Approach for Developing a Dissolution Surrogate

In this case study, a predictive *in vitro* dissolution model was used to establish clinically relevant in-process controls for an amorphous solid dispersion IR drug product (84). Due to the low solubility of the drug substance (BCS class II), hot melt extrusion was selected for processing in order to convert the crystalline form to a more soluble/bioavailable amorphous form. The milled extrudate is blended with excipients and compressed to yield six dose strengths in total during development (four marketed). The dose strengths were all formulated as dose weight multiples, but for differentiating purposes, various tablet sizes and shapes were utilized. Due to the number of dose strengths, an approach based on image-independent properties (i.e., solid fraction, tensile strength) was used throughout development, including efforts to establish the correlation between dissolution and tablet properties.

As outlined herein and aligned with science- and risk-based process development principles, CQAs summarized in the QTPP were linked to material attributes and process parameters. Regarding the CQA of dissolution, an understanding of the dissolution mechanism was necessary in order to understand the impact of material attributes and process parameters on the dissolution results. Referring to Table I, the mechanism for this drug product is tablet disintegration (erosion) followed by the solubilization of the hot melt extrudate particles. The extrudate particle size and crystalline content could theoretically have an impact on dissolution; however, utilization of the established manufacturing controls for the hot melt extrusion and milling steps ensures no meaningful impact examined via drug product testing. Therefore, the rate of tablet disintegration for this product determines the overall dissolution result.

**Table I:** Dissolution Mechanism Summary for an Amorphous Solid Dispersion IR Drug Product with Surrogate Tests Listed for Attributes that Impact Dissolution. Within Manufacturing Controls, Tablet Disintegration Determines Overall Dissolution Rate: %Dissolved(t) = f(hardness, moisture, shape)

Dissolution step	Attribute controlling dissolution step	Impact on dissolution profile?	Surrogate test(s)		
Tablet disintegration	Tablet hardness ➔ Porosity	Yes	Hardness	Disintegration	Solid fraction
	Tablet moisture ➔ Porosity	Yes	Water Activity		
	Tablet shape	Yes	Dimensional measurement		
Particle dissolution	Particle size	Yes, but only for PSD values outside of specification range	Particle size		
	Crystalline content	Yes, but form conversion not observed	XRD, Raman		

An important parameter in tablet compaction, solid fraction is defined as the ratio of the apparent density of the tablet to the true density and is also often referred to as the relative density of the tablet. The apparent density of a tablet is the mass of the tablet divided by the tablet volume. Therefore, the solid fraction, or relative density, can be defined as follows:

2 $$ SF=\frac{\frac{mass}{V_{apparent}}}{\rho_{true}} $$

Theoretically, one minus the solid fraction of the tablet describes the total percentage of open space or porosity in the tablet. Compressed tablets typically range from 0.80 to 0.95 in solid fraction, with 1.0 meaning the apparent density of the tablet equals the true density. Therefore, a solid fraction of 1.0 is the most a tablet could possibly be compressed.

During development, it was determined that the following mechanisms impacted dissolution: (1) changes in compression force and the resulting tablet density and compressive strength (breaking force); (2) moisture uptake by tablets on stability at increased %RH conditions. Tablets compressed to a higher breaking force and density showed slower dissolution profiles. Faster dissolution profiles were seen upon lower initial compression (softer tablets) as well as on stability due to an uptake of moisture. Therefore, due to the sensitivity of disintegration and dissolution to tablet properties, traditional methods of setting in-process compression controls (i.e., resulting comparable dissolution profiles, f_2_ ≥ 50) would have resulted in a narrow operating range that is unrealistic to routinely achieve in a production environment.

The relationship between image-independent properties (solid fraction, tensile strength) and the disintegration/dissolution response was investigated during development. It was found that the tablet density or solid fraction of the tablet was able to account for both mechanisms of dissolution rate changes (tablet properties and moisture). While tensile strength was able to determine the impact of compression on dissolution, moisture absorption results in swelling of the tablet but only in slight changes in tablet hardness. As such, tablet density was able to better correlate the moisture changes in the tablet with the dissolution response. Therefore, decreased solid fraction, whether formed through lower compression or stability-related changes in the tablet, leads to a decreased disintegration time. Also, since solid fraction is a tablet image-independent property, the results were able to be bridged across dose strengths.

**Fig. 7:**
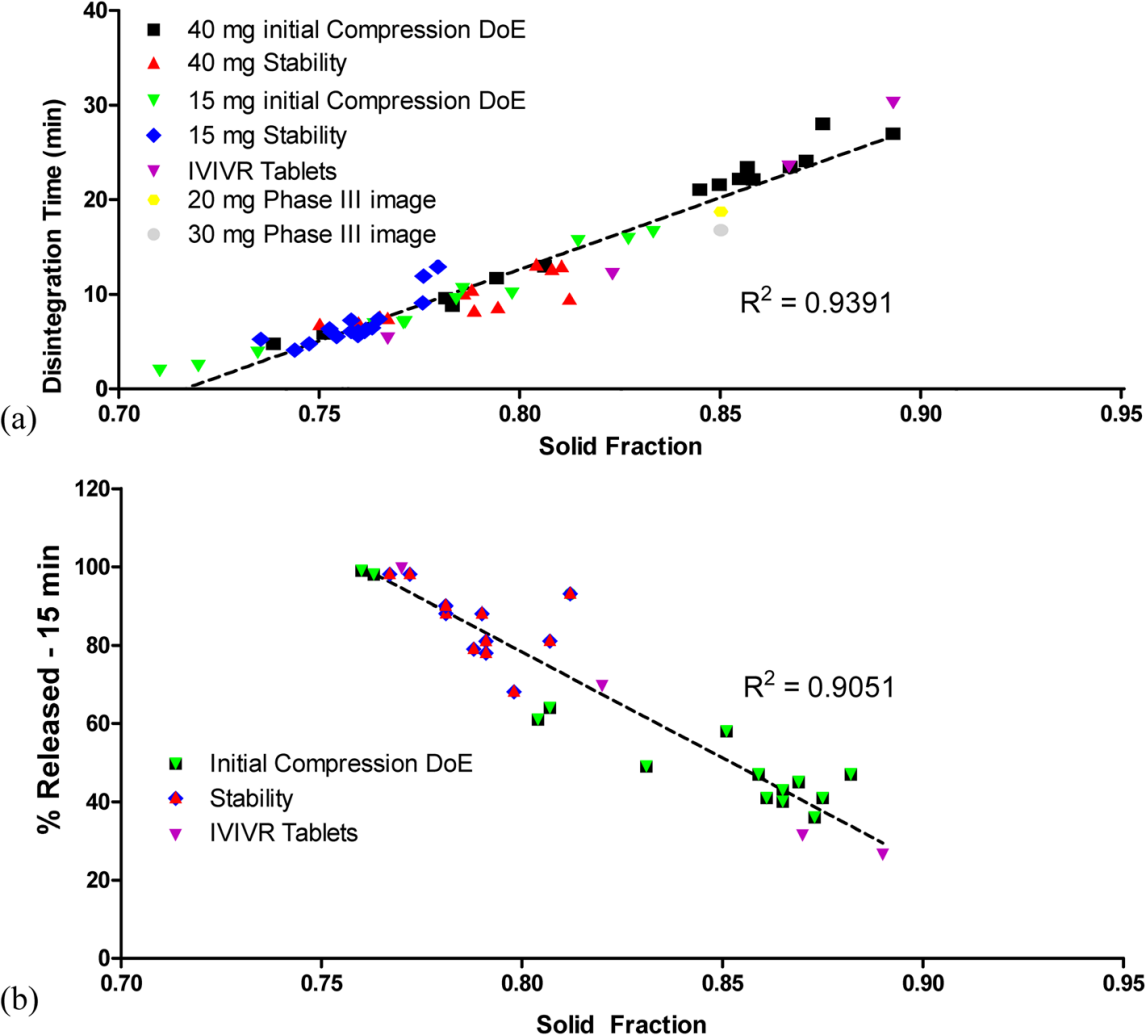
Tablet solid fraction correlations: **a** with disintegration for a range of tablets with different potencies over different compression ranges and exposed to various relative humidity levels (10% RH to 50% RH), and **b** with % dissolved at 15 min from IVIVC study (red), from DoE studies compressed to different compression forces (black), and from tablets exposed to relative humidity levels 10% RH to 50% RH (green)

Figure 7a shows the linear relationship that was established between the tablet solid fraction and disintegration time across dose strengths, compression conditions, and stability conditions. Regardless of whether disintegration was affected by compression force or a change in tablet moisture, the same linear relationship exists between solid fraction and disintegration time. A linear relationship was also established between disintegration and % dissolved at 15 min. Based on the linear relationship of solid fraction with disintegration and the linear relationship of disintegration with % dissolved at 15 min, it is possible to create a linear relationship directly between solid fraction and % dissolved at 15 min. Similar to the solid fraction-disintegration relationship established in Fig. 7a, a linear relationship was established for solid fraction and % dissolved at 15 min (Fig. 7b).

In order to determine how the tablet properties impact clinical pharmacokinetics, a relative bioavailability (BA) study was conducted to compare the pharmacokinetics of tablets manufactured using different compression forces. Based on the results from the BA study, development of a level C IVIVC for disintegration time and multiple level C IVIVC for dissolution was explored. Detailed results of the IVIVC study have been previously published (85). The IVIVC analysis focused on the relationships between dissolution or disintegration and maximum drug concentration in plasma following oral dosage (C_max_). The analysis established a range where the changes in C_max_ are expected to result in equivalent bioperformance (safe space). This range can then be translated into a compression range for the tablets based on the relationship established between disintegration/dissolution and tablet properties.

Tensile strength was chosen as the image-independent property for bridging of the IVIVC results for several reasons: (1) tensile strength allows for better line of sight to the compression in-process controls since hardness is typically used in a production environment; (2) the compression in-process controls do not need to take into account swelling of the tablets that the stability mechanism required. Tensile strength is a function of tablet hardness, thickness, and tablet size, all of which are parameters easily measured on the manufacturing floor. Therefore, the results obtained for tensile strength ranges based on dissolution were also used to set in-process controls for tablet hardness based on the relationship between tablet hardness and tensile strength. Of the dissolution points considered, the 15-min time point was selected because the data resulted in a more discriminating linear relationship between tablet tensile strength and dissolution than other time points; however, a correlation to other dissolution time points or to parameters based on nonlinear regression fits could also be established.

**Fig. 8:**
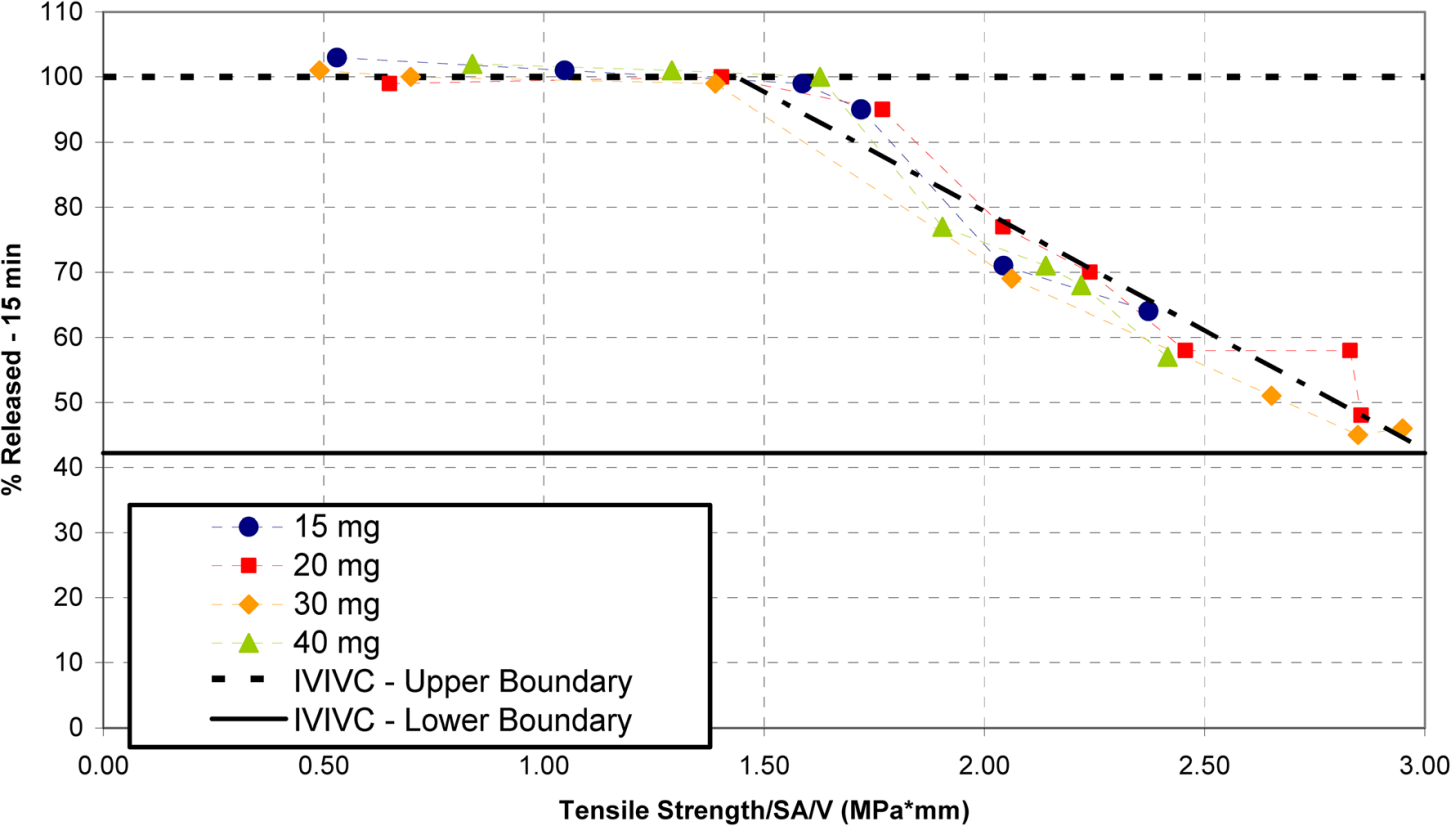
Percent dissolved at 15 min *versus* tablet tensile strength for all dosages—normalized for surface area to volume ratio

The erosion rate of the tablet is a function of the surface area to volume (SA/V) ratio of the image, with the smaller images releasing at a faster rate than the larger dosages at a given tensile strength. When normalizing for the SA/V ratio for each image, it was possible to develop an image-independent relationship between dissolution and normalized tensile strength, as shown in Fig. 8.

Based on the fits shown in Fig. 8, the lower and upper tensile strength limits for each image were able to be calculated based on the intersection of the linear fits with the upper and lower IVIVC/safe space boundaries. Additional considerations such as tablet friability were used to refine the lower tensile strength value for each image. The in-process control limits of tablet hardness were then calculated for each image, resulting in a wider average hardness window that can be reproducibly targeted during production. In summary, the given predictive dissolution model yielded clinically relevant in-process controls for compression that predict with high confidence that every batch within this control space would pass dissolution specification.

**Fig. 9:**
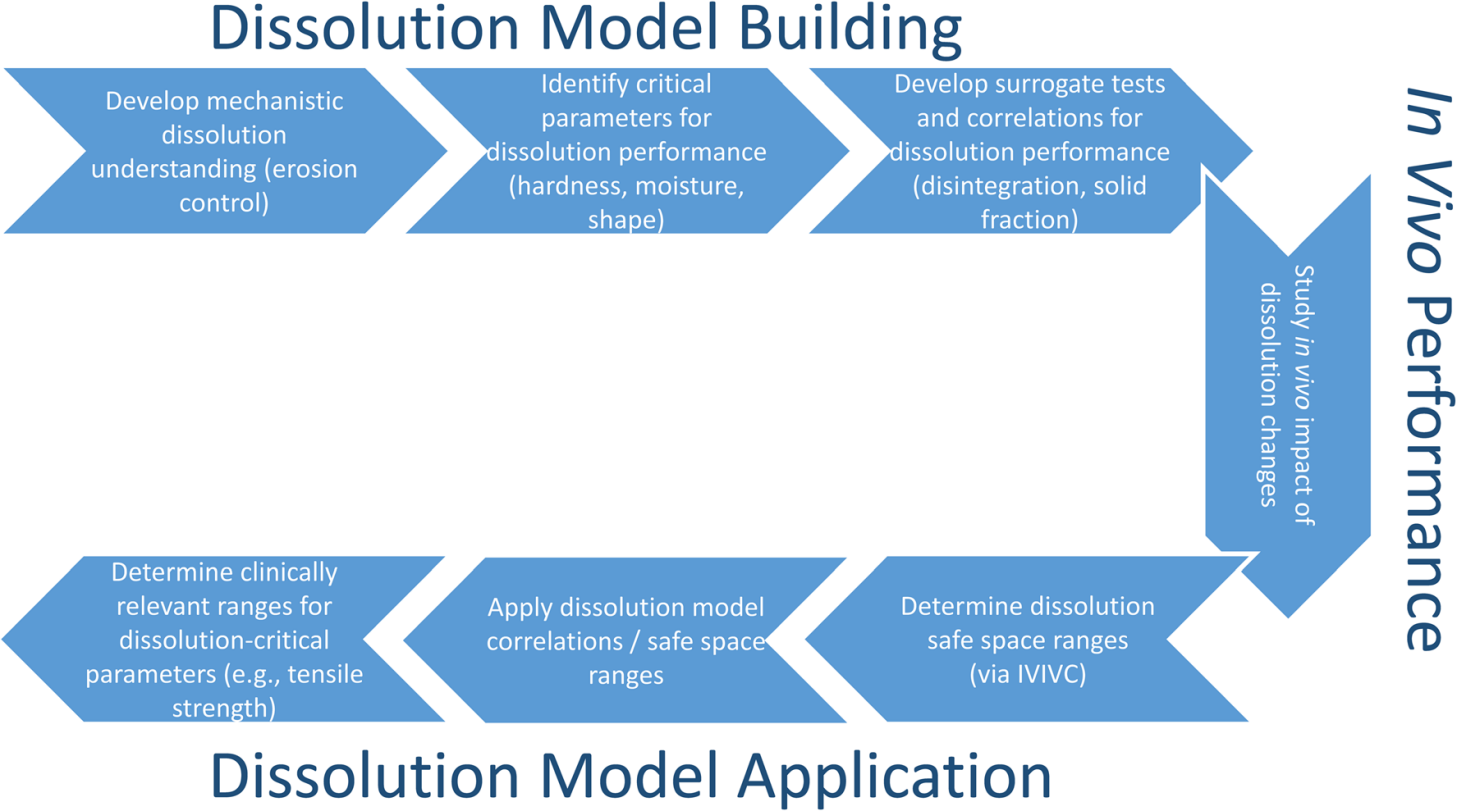
Strategy to build and apply dissolution modeling for case study 3.2 to enable clinical relevance

As demonstrated in the flowchart in Fig. 9, developing a first-principles dissolution understanding and appropriate surrogate tests enabled identification of all critical parameters that impact dissolution performance. More importantly, this made it possible to translate a dissolution safe space into clinically relevant process ranges and controls for parameters such as compression and moisture to ensure consistent *in vivo* performance.

### Dissolution Modeling for RTRt

Dissolution is an integral part of RTRt for both batch and continuous manufacturing, the latter of which plays a key role in enabling the modernization of pharmaceutical manufacturing. Key regulatory concepts for continuous manufacturing, including quality risk management, batch definition, control strategy, process monitoring and control, RTRt, data processing and management, and process validation/verification, have been outlined (85,86). A control strategy is required to ensure a continued state of control throughout the entire operation and to segregate nonconforming materials. Continuous manufacturing offers an opportunity for utilizing real-time data. The implementation of PAT tools (including multivariate tools for design, data acquisition and analysis, process analyzers, and process control tools) helps to ensure an appropriate level of risk control. The use of these tools supports RTRt (although RTRt is not a regulatory requirement for continuous manufacturing) (85,87). There are several examples of implementation of RTRt approaches for dissolution in continuous direct compression (CDC) processes (59,64). The use of predictive dissolution modeling to enable RTRt intends to leverage data collected in-line throughout the CDC manufacturing process (such as drug concentration, tablet hardness, weight, and PSD) to predict a dissolution profile.

Hernandez *et al.* (59) predicted dissolution of tablets with different levels of strain (shear) using NIRS in combination with multivariate data analysis. Tablets produced with different strain levels were measured using NIRS. Spectra were obtained in diffuse reflectance mode and pretreated with baseline correction to maintain the physical and chemical information of the tablets. Dissolution profiles were obtained using USP Apparatus 2 with a reference method. PCA was used to study the sources of variation in the spectra obtained. A PLS calibration model was developed and validated to predict dissolution of tablets with different levels of strain. The study showed that the applied strain affects the dissolution behavior of tablets with similar chemical composition and compaction force. Pawar *et al.* (64) presented a method for predicting dissolution profiles of directly compressed tablets for a fixed sustained release formulation manufactured in a CDC system. A model for predicting dissolution profiles was developed using a fractional factorial experimental design. Four variables (API concentration, blender speed, feed frame speed, and compaction force) were included. The tablets thus obtained were scanned at-line in transmission mode using NIRS. The dissolution profiles were described using two approaches, a model-independent “shape and level” method, and a model-dependent approach based on a Weibull distribution. Multivariate regression was built between the NIRS scores as the predictor variables and the dissolution profile parameters as the response. The model successfully predicted the dissolution profiles of the individual tablets manufactured at the targeted set-point.

Colon *et al.* (88) summarized several approaches for dissolution model development: (1) a model based on measurements and attributes for the tablet formulation only (e.g., using NIRS to obtain API concentration in addition to using weight, hardness, and thickness measurements from a tablet tester); (2) a model based on material properties for both the tablet and pre-tableting formulation (blend or granulation) attributes such as PSD and/or water content, and (3) a model built with tablet data, material attributes going into the tablet (e.g., granule particle size), and process parameters (e.g., compression force). Each approach has benefits and challenges. For all cases, the model needs to comply with regulatory requirements related to method validation and lifecycle management.

A case study showcasing Colon’s second approach has been presented (89). The first step was to determine the dissolution rate (Z) from reference dissolution profiles. This information was used for the selection of the appropriate dissolution fit model to best describe the kinetics and mechanism of the dissolution rates (e.g., Noyes-Whitney, Weibull, or a hybrid model), as well as the rate factor (Z) and the plateau (p). Once the best-fit model was selected, the relationship between the input variables and the “rate” variable in the fit model was established. Selection of the variables for inclusion into the predictive model should be based upon their statistical significance to the prediction of dissolution or upon their known physical impact to dissolution. In the case study, the measured attribute data were granule PSD, the final blend API and water content, and the core tablet weight, hardness, and thickness. For routine use, dissolution rate was then predicted and used to construct a full dissolution profile.

### Existing Challenges and Perspective

Although there are successful case studies from industry and academia of using predictive dissolution models for early development, in-process control, and CM RTRt or batch release, challenges to predictive dissolution modeling remain that should be addressed in order to continue the advancement of this field. The following areas present some of the most pressing challenges and opportunities for advancement.

1. Although this paper is based primarily on and intended for development of new IR drug products, the guidelines for the use of empirical and first-principles approaches to model and simulate dissolution are expected to apply to legacy products and to sustained- and controlled-release formulations, or even non-oral solid dosage forms (such as transdermal patch), with appropriate considerations of the additional complexities of the formulation and route of administration.
2. As predictive dissolution modeling increases in prevalence in pharmaceutical practice, a strong need arises for increased dialogue between industry practitioners and regulatory authorities on implementing predictive dissolution models for RTRt in CM and of acceptance criteria for model use in submission. Sharing information with regulatory agencies is considered to be of utmost importance in order to facilitate implementation and potential harmonization of regulatory expectations across different geographical territories.
3. While commercial dissolution modeling software exists, its applications and customizability for *in vitro* dissolution are limited. Typical dissolution modeling packages are built around diffusive flux from particle surface and are primarily focused on *in vivo* dissolution simulation or parameter estimation. No commercial software is currently capable of simulating some of the more complex dissolution aspects of modeling (e.g., correct accounting for surface pH, separation of granule and API modeling, API form change, separation of surface kinetics and diffusive and convective fluxes) or of establishing RTRt protocols. Thus, industrial researchers devote time and effort to developing custom models, often duplicating previous efforts by colleagues across the industry. Greater communication between pharmaceutical companies and software developers may be beneficial to expand the use and acceptability of *in vitro* dissolution modeling.
4. PBPK simulation and modeling in pharmaceutical product development is also useful for reducing development cost and to aid decision-making, independent of or in tandem with dissolution modeling. The combination of the two can leverage the understanding of the drug obtained from one approach to strengthen the models of the other, such as for the use of virtual bioequivalence studies (69). PBPK modeling can be done in lieu of empirical *in vivo* or *in vitro* experiments. For BCS class II and IV compounds, it is possible to leverage late-phase *in vivo* PK data to adjust the *in vitro* dissolution method in order to develop a clinically relevant dissolution method and to deploy it as a QC method for manufacturing environment. However, the clinical relevance of an *in vitro* dissolution method is highly dependent on whether the absorption of the compound is dissolution rate limited or not. For compounds where the absorption rate is not dissolution rate limited, the PBPK simulation could be used in lieu of experiments to justify the “safe space” and its boundary. If the absorption of the compound is dissolution rate limited, the PBPK simulation and modeling can be a powerful tool to enable scientists to establish clinical relevance even in early-phase dissolution method development. It is fair to admit that PBPK modeling requires comprehensive knowledge of the compound’s solubility, permeability, GI transit, and potential BA studies to develop a meaningful model. However, given the resource-consuming nature of human PK studies, it is expected to be in the best interest of pharmaceutical scientists to continue pushing the boundary of PBPK modeling with the intention of leveraging IVIVC (if possible) as early as possible in pharmaceutical product development to accelerate development and to reduce its cost.

## CONCLUSION

**Fig. 10:**
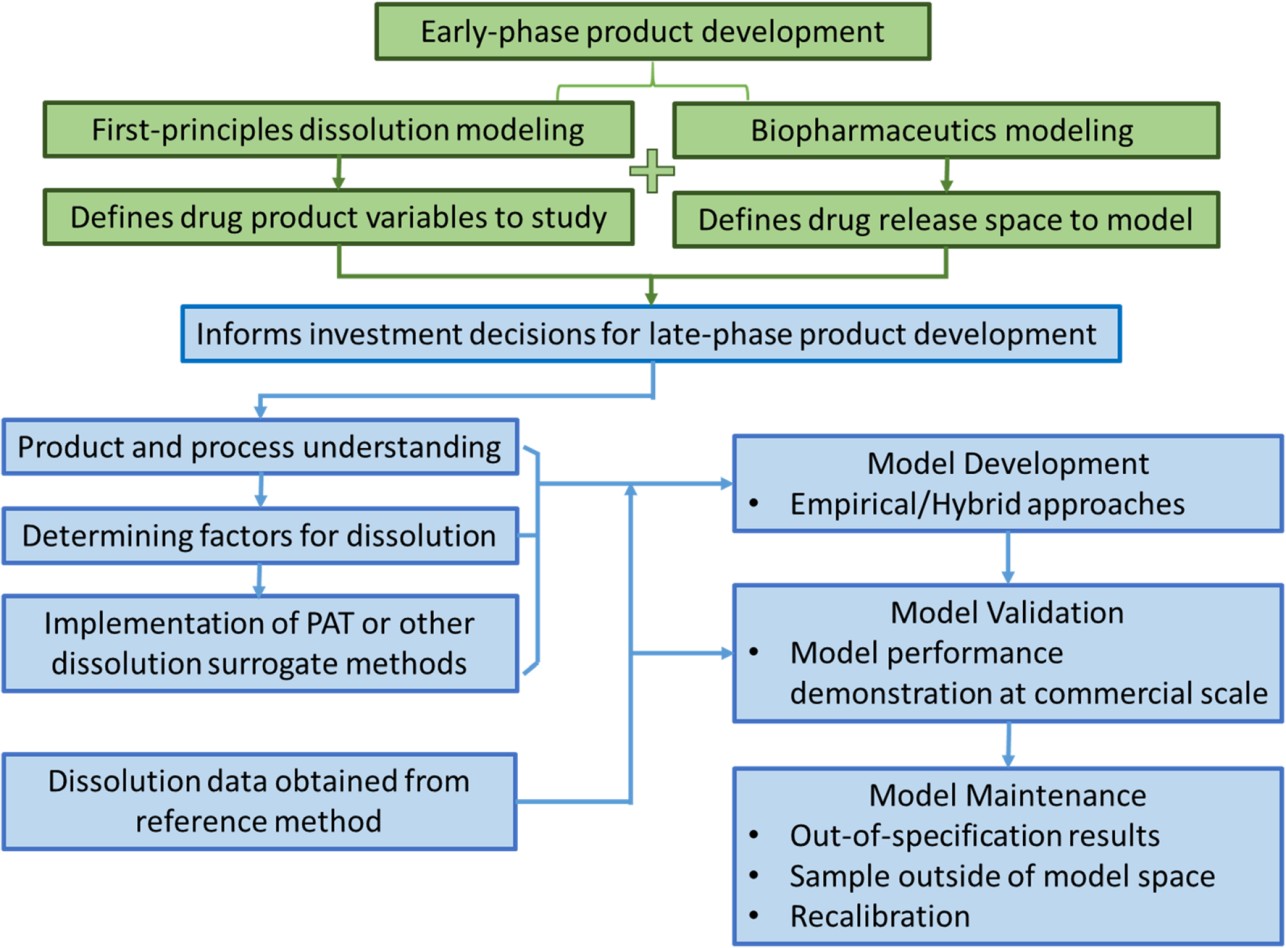
General roadmap for predictive *in vitro* dissolution model development

The paper illustrates a general strategy for predictive *in vitro* dissolution model development and a general roadmap (Fig. 10) for its implementation for enhanced product understanding, robust control strategy, batch release testing, and flexibility for post-approval changes. The selection among various modeling approaches based on product and process understanding is demonstrated at different phases of drug development. Early in the research and development process, when little data and process understanding is available, first-principles models can be used to provide guidance for formulation and process development. As greater amounts of data and understanding are generated, a first-principles-based and data-driven empirical approach becomes affordable for linking material attributes and process conditions to the drug product dissolution profile in order to enable a predictive dissolution model for batch or real-time release. Post-approval changes can utilize the same framework, relying on first principles to understand the effect of the change and on existing or extended empirical dissolution models for product release with minimal additional experimental burdens.

## Abbreviations

API: Active pharmaceutical ingredient
BA: Bioavailability
BCS: Biopharmaceutics Classification System
CDC: Continuous direct compression
cGMP: Current good manufacturing practices
CMA: Critical material attributes
Cmax: Maximal plasma concentration
CMC: Chemistry, manufacturing, and controls
CPP: Critical process parameter
CQA: Critical quality attribute
DoE: Design of experiments
DS-A: Drug substance A
FaSSIF: Fasted-state simulated intestinal fluid
FDA: Food and Drug Administration
GI: Gastrointestinal
ICH: International Conference on Harmonisation of Technical Requirements for Registration of Pharmaceuticals for Human Use
IQ: International Consortium for **I**nnovation and **Q**uality in Pharmaceutical Development
IR: Immediate release
IVIVC: *In vivo*-*in vitro* correlation
MR: Modified release
NDA: New drug application
NIRS: Near-infrared spectroscopy
PAT: Process analytical technology
PBPK: Physiology-based pharmacokinetics
PCA: Principal components analysis
PK: Pharmacokinetics
PLS: Partial least squares
PSD: Particle size distribution
QC: Quality control
QTPP: Quality target product profile
RTRt: Real-time release testing
SA/V: Surface area to volume ratio
USP: United States Pharmacopoeia
XRD: X-ray diffractometry
